# A Retrospective Comparison Trial Investigating Aggregate Length of Stay Post Implementation of Seven Enhanced Recovery After Surgery (ERAS) Protocols between 2015 and 2022

**DOI:** 10.3390/jcm13195847

**Published:** 2024-09-30

**Authors:** Rebecca N. Blumenthal, Andrew R. Locke, Noah Ben-Isvy, Muneeb S. Hasan, Chi Wang, Matthew J. Belanger, Mohammed Minhaj, Steven B. Greenberg

**Affiliations:** 1 Department of Anesthesiology, Critical Care, and Pain Medicine, Endeavor Health, Evanston, IL 60201, USA; 2 Department of Anesthesiology and Critical Care, Pritzker School of Medicine, University of Chicago, Chicago, IL 60637, USA; 3 Department of Biostatistics, Endeavor Health, Evanston, IL 60201, USA

**Keywords:** anesthesia, Enhanced Recovery After Surgery, perioperative medicine

## Abstract

(1) **Introduction**: Enhanced Recovery After Surgery (ERAS) protocols can create a cultural shift that will benefit patients by significantly reducing patient length of stay when compared to an equivalent group of surgical patients not following an ERAS protocol. (2) **Methods**: In this retrospective study of 2236 patients in a multi-center, community-based healthcare system, matching was performed based on a multitude of variables related to demographics, comorbidities, and surgical outcomes across seven ERAS protocols. These cohorts were then compared pre and post ERAS protocol implementation. (3) **Results**: ERAS protocols significantly reduced hospital length of stay from 3.0 days to 2.1 days (*p* <0.0001). Additional significant outcomes included reductions in opioid consumption from 40 morphine milligram equivalents (MMEs) to 20 MMEs (*p* <0.001) and decreased pain scores on postoperative day zero (POD 0), postoperative day one (POD 1), and postoperative day two (POD 2) when stratified into mild, moderate, and severe pain (*p* <0.001 on all three days). (4) **Conclusions**: ERAS protocols aggregately reduce hospital length of stay, pain scores, and opioid consumption.

## 1. Introduction

Enhanced Recovery After Surgery (ERAS) protocols are evidence-based, multidisciplinary and collaborative approaches to perioperative care that provide transformative plans for minimizing pain, reducing opioid administration, expediting patient recovery, and decreasing perioperative complications and hospital length of stay [1,2,3].

ERAS initiatives are important in providing safe care and increasing patient satisfaction in hospital systems [1,2,3]. Successful implementation involves collaboration between anesthesiology, surgery, nursing teams, and many other hospital professionals who agree upon and implement a number of perioperative interventions into an evidence-based protocol [4,5,6,7]. ERAS programs start with patient education. In the preoperative period, education targets expectations about surgery and encourages patient participation in their care [8,9]. Preoperative education also emphasizes the importance of preoperative hydration, focusing on up-to-date fasting guidelines, and describes the administration of non-opioid analgesics throughout the perioperative period [10,11,12]. Intraoperatively, providers administer multimodal non-opioid analgesics and multimodal nausea and vomiting prophylactics, maintain normothermia and euvolemia, and administer regional anesthetic blocks with local anesthetics to improve and reduce pain [11,12,13,14]. In the postoperative setting, early nutrition and mobilization, continuation of multimodal non-opioid analgesics, and early removal of drains, catheters, and tubes aid in further expeditious recovery [11,12,13].

There are numerous studies that have been published previously describing success of individual ERAS protocols in multiple surgical subspecialties both in academic and community-based practices across the United States [15,16,17,18,19,20,21,22,23]. There are also several meta-analyses published of ERAS programs in a variety of surgical subspecialties in community-based and academic institutions across the world, which suggest a reduction in length of stay, opioid use, complications, and cost [1,2,3,24,25]. However, none provide a pooled analysis from one entire community-based healthcare system.

In this retrospective study, the investigators compared the ERAS pre-implementation and post-implementation aggregate length of stay, maximum pain scores, and total opioid usage (MMEs) among seven surgical procedures. We hypothesize that in a multi-center community-based health system, the aggregate length of stay among seven surgical subspecialties would be significantly reduced after ERAS implementation. In addition, we hypothesize that the addition of these customized ERAS protocols in a community healthcare system would result in a reduction in patient pain scores and opioid usage in the postoperative period.

## 2. Materials and Methods

The ERAS program at Endeavor Health (a four-hospital community-based healthcare system in Chicago, IL at the time of the study) was created between 2016 and 2021 and involved seven unique ERAS protocols: colorectal, ventral hernia, implant-based mastectomy, Cesarean section, open abdominal hysterectomy, prostatectomy, and hepatobiliary surgeries. This study received Endeavor Health Institutional Review Board approval, and written informed consent was waived, given the retrospective design.

The Endeavor Health ERAS protocols have three distinct phases during the perioperative period: preoperative, intraoperative, and postoperative. In the preoperative phase, the main interventions include patient education and medical optimization, up-to-date preoperative fasting guidelines and carbohydrate loading, elimination of mechanical bowel preps, multimodal non-opioid analgesic premedications, and thromboembolic and antimicrobial prophylaxis. During the intraoperative phase, there is an emphasis on multimodal, non-opioid analgesics and antiemetics, regional anesthesia techniques are utilized, patients are kept normothermic and euvolemic, and drain, catheter, and nasogastric-tube usage is minimized. The postoperative components focus on early mobilization and ambulation, early nutrition, and continuation of multimodal, non-opioid analgesics.

This study includes data collected pre and post implementation for each individual ERAS protocol. The pre-ERAS cohort in this study consists of patients who underwent surgery approximately one to two years before the implementation of each individual ERAS protocol, while the ERAS cohort consists of patients in each group from July 2021 to June 2022 after all ERAS protocols were implemented (Table 1). Additionally, during the time frame of ERAS data collection, the total volume of surgical patients participating in ERAS protocols at Endeavor was at its highest. Data were derived from an electronic Endeavor Health data warehouse system that facilitated individual outcome-based study endpoints. Chart review was relied upon to validate individual outlier data points. Pre-ERAS implementation data were collected retrospectively, and ERAS data were collected prospectively at the time of enrollment in the program.

Patients were matched based on the following variables: type of surgery, age, gender, BMI, ASA class, kidney disease *, liver disease *, chronic opioid use/abuse *, chronic pain *, cardiovascular disease *, respiratory disease *, neurologic disease *, procedure time, ICU admission, return to OR on same admission, return to OR within 30 days, discharge location, and hospital readmission.

The study’s primary outcome was the comparative aggregate length of stay between the pre-ERAS and ERAS cohorts. The secondary outcomes comparing the aggregate of the two cohorts were as follows: (1) total postoperative opioid consumption in the hospital measured in morphine milligram equivalents (MMEs), (2) the proportion of patients that received no postoperative opioids, (3) maximum pain score on postoperative day 0 (POD 0), (4) maximum pain score on POD 1 (5) maximum pain score on POD 2 and (6) the number of patients who met mild pain scores (0–3/10) on postoperative days 0–2. Pain scores are recorded in the EMR by use of a numeric pain scale of 0–10.

* Pulled from the EMR based on ICD10 codes (Appendix A).

### Statistical Analysis

Patient characteristics were compared between pre-ERAS and ERAS groups using Chi-square test for categorical variables and Wilcoxon rank sum test for continuous variables, and were presented as median with interquartile range. A propensity score was estimated by fitting a logistic regression model which adjusted for type of surgery, gender, age, BMI, ASA class, kidney disease *, liver disease *, chronic opioid use/abuse *, chronic pain *, cardiovascular disease *, respiratory disease *, neurologic disease *, procedure time, ICU admission, return to OR on same admission, return to OR within 30 days, discharge location, and hospital readmission. One-to-one pair matching between the two groups was performed by nearest-neighbor matching without replacement. Covariate balances before and after matching were checked by comparison of standardized mean difference (SMD). A SMD < 0.1 was considered to indicate a proper balance between the two groups. All tests were 2-tailed and *p* < 0.05 was considered to be statistically significant. All analyses were performed with the SAS statistical package (version 9.4, SAS Institute, Cary, NC, USA).

## 3. Results

This retrospective study analyzed a total of 2236 patients who underwent seven different surgical specialty cases during the time intervals of interest. A total of 798 surgical patients were included in the pre-ERAS group, a cohort of patients pooled from the year prior to implementation of each individual ERAS protocol. Additionally, a total of 1438 patients were included in the ERAS group.

After propensity score matching based on demographic and clinical characteristics, a total of 1324 patients were included in the analysis between the pre-ERAS (*n* = 662) and ERAS (*n* = 662) groups. Table 2 suggests that all demographic and clinical characteristics were not statistically significant between groups. In addition, there were no differences in discharge location or hospital readmission rates.

Table 3 outlines numerical and distributional differences in length of stay between pre-ERAS and ERAS groups. The median length of stay in the pre-ERAS group was 3.0 days compared to 2.1 days in the ERAS group (*p* < 0.0001). Additionally, based on distributional differences in length of stay (*p* < 0.0001), patients were more likely to be discharged in less than 3 days in the ERAS group when compared to pre-ERAS. 

Table 4 demonstrates the ERAS group patients consumed fewer opioids in hospital compared to the pre-ERAS group, as measured by MMEs (40 vs. 20, *p* < 0.0001). Distributional differences in maximum pain scores were found to be statistically significant between the two groups on POD 0 (*p* < 0.0001), POD 1 (*p* < 0.0001), and POD 2 (*p* < 0.0001). An increased number of patients in the ERAS group experienced mild pain (0–3/10) as opposed to moderate or severe pain on POD 0 (*p*< 0.0001), POD 1 (*p* < 0.0001), and POD 2 (*p*< 0.0001) when compared to pre-ERAS patients. Similarly, there was a statistically significant reduction in median pain scores in the ERAS group on POD 0 (7 vs. 6, *p* < 0.0001), POD 1 (6 vs. 5, *p* < 0.0001), and POD 2 (6 vs. 5, *p* = 0.0004).

## 4. Discussion

This retrospective study demonstrates the positive impact of implementation of multiple ERAS protocols at a multi-specialty, community-based hospital system. By investigating seven surgical specialties, this study provides a comprehensive and real-life analysis of the impact of ERAS implementation in aggregate. The pooled ERAS data clearly show a statistically significant reduction in length of stay by approximately one day. This study also suggests a reduction in opioid consumption by 50% and the achievement of more patients who experienced mild pain versus moderate or severe pain on POD 0–3.

A 50% reduction in opioid consumption using the current ERAS techniques appears to be one major step in curbing the opioid-dependence epidemic. Alarmingly, approximately 7% of all surgical patients in the US develop opioid dependence after surgery [26]. Research also suggests that patients who have high requirement for opioids as an inpatient utilize large quantities of opioids after discharge as well [27]. Because of improved perioperative pain techniques, opioid usage was minimized with multiple ERAS protocol implementations in this multi-specialty community-based hospital system. In addition, ERAS patients in this study experienced a subjective reduction in pain in the first three postoperative days, suggesting that patients’ pain was better controlled overall.

Another benefit of development of an ERAS program in multiple surgical subspecialties in a community hospital system is reduction in hospital length of stay. The one-day aggregate reduction in hospital length of stay in this study could mean millions of dollars of savings for hospitals and patients over time. According to the Kaiser Family Foundation, an average cost of a day in the hospital is approximately USD 3000 [28]. If all of the non-ERAS patients in this trial were converted into ERAS patients, we could potentially expect a cost savings of USD 1,986,000 annually. In a healthcare era where value-based care is a priority, this cost savings appears worth the upstart costs of any ERAS program among a variety of surgical specialties.

ERAS protocols have become increasingly recognized as integral components of perioperative care, aiming to enhance patient outcomes while optimizing resource utilization [29]. The primary outcomes of this study align with the broader literature on ERAS protocols, demonstrating significant reductions in hospital length of stay [30,31,32,33,34,35,36], postoperative pain [36,37,38], and opioid consumption [37,38]. These findings underscore the effectiveness of ERAS interventions in promoting enhanced recovery across diverse surgical specialties within a community hospital system setting. The observed decrease in hospital length of stay suggests a more efficient recovery process [30,32,35], minimizing the duration of inpatient care while ensuring appropriate patient analgesia. Moreover, the reduction in opioid consumption in our ERAS patients reflects the successful implementation of multimodal analgesia strategies, including non-opioid analgesics and regional blocks. This aligns with the principles of ERAS aimed at minimizing opioid-related complications and facilitating earlier mobilization and discharge [36,37,38].

While similar studies have explored the impact of ERAS protocols within individual surgical subspecialties in both academic and community-based medical systems [4,6,7,9,14,15,16,17,18,19,20,21,22,23,24,25,29,30,31,32,33,34,35,36,37,38], this study stands out for its focus on the impact of multiple ERAS protocols in a multi-specialty community hospital setting. The aggregate reduction in hospital length of stay, along with the significant reduction in total opioid consumption (MMEs) demonstrates the success that can be achieved by developing and implementing an ERAS program at a community-based hospital system. The consistent reduction in hospital length of stay, opioid consumption, and maximum pain scores on POD 0, POD 1, and POD 2 observed across different surgical specialties reaffirms the effectiveness of ERAS principles in various patient populations and procedures. The implications of this study extend beyond the specific context of the investigated community hospital system, emphasizing the broader applicability of ERAS protocols across various healthcare settings and surgical disciplines. The observed benefits in terms of reduced length of stay, postoperative pain, and opioid consumption underscore the potential for ERAS to enhance resource utilization [29], improve patient outcomes, and mitigate the burden of postoperative complications in a real-world environment. By emphasizing the importance of collaborative, evidence-based perioperative care, this study advocates for the widespread adoption of ERAS protocols as routine practice, irrespective of hospital size or surgical specialty.

It is important to note that creation and rollout of ERAS protocols is a difficult and time-consuming process that presents many challenges for hospital systems. There are a number of roadblocks that may impede successful ERAS implementation, including cost restraints, resource availability, administrative support, a lack of enthusiastic ERAS champions, buy-in from all providers and patients, involved quality managers, and reliable ancillary support. At our institution, the biggest impediments during ERAS development have been resource availability, specifically the need to hire more anesthesia technicians and purchase additional ultrasound equipment to aid in regional blocks, and support from all surgical and anesthesia professionals.

Several limitations warrant consideration when interpreting the findings of this study. First, the impact of external factors such as changes in clinical practice or healthcare policies over the study period could confound the observed associations. In addition, because the data from this study were pooled from 2015 to 2022, changes in the healthcare billing model and economic factors such as inflation limit the ability to accurately assess changes in cost pre and post ERAS implementation. Furthermore, the occurrence of the COVID-19 pandemic during the study period may have introduced variables that were not accounted for in the analysis, potentially influencing perioperative care practices and outcomes. This study was performed in a community-health multiple hospital system, and, therefore, the findings may be different in other parts of the world and other healthcare settings. Lastly, the retrospective nature of the study may have led to undefined biases.

## 5. Conclusions

This retrospective comparison trial highlights the positive impact of ERAS protocols on patient outcomes across multiple surgical specialties within a community hospital system. Despite inherent limitations, the findings of this study contribute to the growing body of evidence supporting the widespread adoption of ERAS protocols as a recognized practice in enhancing surgical recovery and optimizing resource utilization. Understanding which interventions are the most impactful could allow a more focused design of future ERAS protocols. Finally, qualitative studies investigating patient perspectives and experiences following ERAS protocols could provide valuable insights into the patient-centered benefits of enhanced recovery initiatives.

## Figures and Tables

**Table 1:** Data Collection Time Frame.

Protocol	Pre-ERAS Start	Pre-ERAS End	ERAS Start	ERAS End
Colorectal	9/15/2015	9/22/2016	7/1/2021	6/30/2022
Ventral Hernia	9/30/2016	9/20/2017	7/1/2021	6/30/2022
Mastectomy	3/1/2017	2/28/2018	7/7/2021	6/30/2022
Hysterectomy	6/30/2017	9/4/2018	7/2/2021	6/28/2022
C-Section	10/17/2016	4/30/2018	7/1/2021	6/30/2022
Prostatectomy	6/25/2018	6/19/2019	7/12/2021	6/28/2022
Hepatobiliary	9/26/2019	9/23/2020	7/1/2021	6/30/2022

**Table 2:** Cohort Demographics.

Variable	Overall (N = 1324)		Pre-ERAS (*n* = 662)	ERAS (*n* = 662)	
	*n*, (Range)	Median (IQR)	Median (IQR)	Median (IQR)	*p*-Value
**Demographics**					
**ERAS Protocol**					0.255
Colorectal		391 (29.5)	191 (28.9)	200 (30.2)	
Ventral Hernia		49 (3.7)	28 (4.2)	21 (3.2)	
Implant-Based Post-Mastectomy		103 (7.8)	61 (9.2)	42 (6.3)	
Open Abdominal Hysterectomy		175 (13.2)	83 (12.5)	92 (13.9)	
C-Section		218 (16.5)	99 (15.0)	119 (18.0)	
Prostatectomy		266 (20.1)	140 (21.1)	126 (19.0)	
Hpb		122 (9.2)	60 (9.1)	62 (9.4)	
**Patient Gender**					0.571
F		822 (62.1)	406 (61.3)	416 (62.8)	
M		502 (37.9)	256 (38.7)	246 (37.2)	
**AGE**	*n* =1324	60.0 (44.0–69.0)	60.0 (45.0–69.0)	60.0 (43.0–69.0)	0.575
**BMI**	*n* = 1159	27.2 (23.6–31.3)	27.0 (23.5–31.0)	27.5 (23.7–31.7)	0.262
**FINAL ASA RATING**					0.253
1		39 (3.0)	25 (3.8)	14 (2.1)	
2		842 (64.2)	426 (64.8)	416 (63.5)	
3		419 (31.9)	200 (30.4)	219 (33.4)	
4		12 (0.9)	6 (0.9)	6 (0.9)	
**Cardiovascular System Disorder**					0.748
No		1144 (86.4)	574 (86.7)	570 (86.1)	
Yes		180 (13.6)	88 (13.3)	92 (13.9)	
**Renal System Disorder**					0.690
No		1214 (91.7)	609 (92.0)	605 (91.4)	
Yes		110 (8.3)	53 (8.0)	57 (8.6)	
**Hepatic System Disorder**					0.822
No		1240 (93.7)	619 (93.5)	621 (93.8)	
Yes		84 (6.3)	43 (6.5)	41 (6.2)	
**Neurological System Disorder**					0.563
No		1321 (99.8)	660 (99.7)	661 (99.8)	
Yes		3 (0.2)	2 (0.3)	1 (0.2)	
**Opioid Use/Abuse Disorder**					0.910
No		1322 (99.8)	661 (99.8)	661 (99.8)	
Yes		2 (0.2)	1 (0.2)	1 (0.2)	
**Pain Disorder**					0.675
No		1226 (92.6)	611 (92.3)	615 (92.9)	
Yes		98 (7.4)	51 (7.7)	47 (7.1)	
**Respiratory System Disorder**					0.832
No		1228 (92.7)	615 (92.9)	613 (92.6)	
Yes		96 (7.3)	47 (7.1)	49 (7.4)	
**Operative Outcomes**					
**Procedure Time (Minutes)**	*n* = 1305	154.0 (97.0–201.0)	153.0 (100.0–197.0)	154.5 (93.0–205.5)	0.789
**ICU Admission**					0.817
No		1305 (98.6)	653 (98.6)	652 (98.5)	
Yes		19 (1.4)	9 (1.4)	10 (1.5)	
**Return to OR Same Admission**					0.428
No		1298 (98.0)	651 (98.3)	647 (97.7)	
Yes		26 (2.0)	11 (1.7)	15 (2.3)	
**Return to OR within 30 days**					0.413
No		1318 (99.5)	658 (99.4)	660 (99.7)	
Yes		6 (0.5)	4 (0.6)	2 (0.3)	
**Discharge Location**					0.727
Discharged With Home Health		82 (6.2)	40 (6.0)	42 (6.3)	
Expired		1 (0.1)	1 (0.2)	0 (0)	
Home Or Self Care		1196 (90.3)	597 (90.2)	599 (90.5)	
Hospice Med Facility		1 (0.1)	0 (0)	1 (0.2)	
Hospice/Home		0 (0)	0 (0)	0 (0)	
Intermediate Care Facility		0 (0)	0 (0)	0 (0)	
Iv Therapy Provider		1 (0.1)	1 (0.2)	0 (0)	
Left Against Medical Advice		0 (0)	0 (0)	0 (0)	
Nursing Home		1 (0.1)	0 (0)	1 (0.2)	
Rehab Facility		2 (0.2)	1 (0.2)	1 (0.2)	
Skilled Nursing Facility		40 (3)	22 (3.3)	18 (2.7)	
**Hospital Readmission**					0.813
No		1248 (94.3)	625 (94.4)	623 (94.1)	
Yes		76 (5.7)	37 (5.6)	39 (5.9)	

**Table 3:** Length of Stay.

Variable	Overall (*n* = 1324)		Pre-ERAS (*n* = 662)	ERAS (*n* = 662)	
	*n*, (Range)	Median (IQR)	Median (IQR)	Median (IQR)	*p*-Value
**Length of Stay**	*n* = 1324	2.7 (1.1–3.9)	3.0 (1.5–4.1)	2.1 (1.1–3.2)	<0.0001
0 to 1		265 (20.0)	116 (17.5)	149 (22.5)	<0.0001
1.1 to 3		567 (42.8)	240 (36.3)	327 (49.4)	
3.1 to 5		336 (25.4)	215 (32.5)	121 (18.3)	
5+		156 (11.8)	91 (13.7)	65 (9.8)	

**Table 4:** Opioid Consumption and Pain Scores.

Variable	Overall (*n* = 1324)		Pre-ERAS (*n* = 662)	ERAS (*n* = 662)	
	*n*, (Range)	Median (IQR)	Median (IQR)	Median (IQR)	*p*-Value
**Opioid Consumption (MMEs)**	*n* = 908	30.0 (10.0–84.5)	40.0 (15.0–117.5)	20.0 (10.0–52.5)	<0.0001
**Max Pain Score POD 0**	*n* = 1215	7.0 (5.0–8.0)	7.0 (5.0–8.0)	6.0 (4.0–8.0)	<0.0001
0 to 3		172 (14.2)	62 (9.8)	110 (18.9)	<0.0001
4 to 6		417 (34.3)	208 (32.9)	209 (35.8)	
7 to 10		626 (51.5)	362 (57.3)	264 (45.3)	
**Max Pain Score POD 1**	*n* = 1230	5.0 (4.0–7.0)	6.0 (4.0–7.0)	5.0 (3.0–7.0)	<0.0001
0 to 3		270 (22.0)	109 (17.0)	161 (27.4)	<0.0001
4 to 6		522 (42.4)	287 (44.6)	235 (40.0)	
7 to 10		438 (35.6)	247 (38.4)	191 (32.5)	
**Max Pain Score POD 2**	*n* = 897	5.0 (4.0–7.0)	6.0 (4.0–7.0)	5.0 (3.0–7.0)	0.0004
0 to 3		217 (24.2)	88 (18.2)	129 (31.2)	<0.0001
4 to 6		387 (43.1)	225 (46.5)	162 (39.2)	
7 to 10		293 (32.7)	171 (35.3)	122 (29.5)	

## Data Availability

De-identified data can be obtained with reason and institutional approval by contacting the corresponding author.

## Appendix A

ICD 10 Codes:

Kidney

E10.22	Type 1 diabetes mellitus with diabetic chronic kidney disease
E10.29	Type 1 diabetes mellitus with other diabetic kidney complication
E11.22	Type 2 diabetes mellitus with diabetic chronic kidney disease
E11.29	Type 2 diabetes mellitus with other diabetic kidney complication
E13.22	Other diabetes mellitus with diabetic chronic kidney disease
E13.29	Other diabetes mellitus with other diabetic kidney complication
I12.0	Hypertensive chronic kidney disease with stage 5 chronic kidney disease or ESRD
I12.9	Hypertensive chronic kidney disease with stage 1-4/unspecified chronic kidney
I13.0	Hypertensive heart and chronic kidney disease with heart failure and stage 1-4/unspecified chronic kidney
I13.10	Hypertensive heart and chronic kidney disease without heart failure, with stage 1-4/unspecified chronic kidney
I13.11	Hypertensive heart and chronic kidney disease without heart failure, with stage 5 chronic kidney/ESRD
I13.2	Hypertensive heart and chronic kidney disease with heart failure and with stage 5 chronic kidney/ESRD
I15.0	Renovascular hypertension
I15.1	Hypertension secondary to other renal disorders
I75.81	Atheroembolism of kidney
N17.0	Acute kidney failure with tubular necrosis
N17.1	Acute kidney failure with acute cortical necrosis
N17.2	Acute kidney failure with medullary necrosis
N17.8	Other acute kidney failure
N17.9	Acute kidney failure, unspecified
N18.1	Chronic kidney disease, stage 1
N18.2	Chronic kidney disease, stage 2 (mild)
N18.3	Chronic kidney disease, stage 3 (moderate)
N18.30	Chronic kidney disease, stage 3 unspecified
N18.31	Chronic kidney disease, stage 3a
N18.32	Chronic kidney disease, stage 3b
N18.4	Chronic kidney disease, stage 4 (severe)
N18.5	Chronic kidney disease, stage 5
N18.6	End-stage renal disease
N18.9	Chronic kidney disease, unspecified
N19	Unspecified kidney failure
N26.1	Atrophy of kidney (terminal)
N26.2	Page kidney
N26.9	Renal sclerosis, unspecified
N27.0	Small kidney, unilateral
N27.1	Small kidney, bilateral
N27.9	Small kidney, unspecified
N28.0	Ischemia and infarction of kidney
N28.1	Cyst of kidney, acquired
N28.81	Hypertrophy of kidney
N28.89	Other specified disorders of kidney and ureter
N28.9	Disorder of kidney and ureter, unspecified
N29	Other disorders of kidney and ureter in diseases classified elsewhere
N99.0	Postprocedural (acute) (chronic) kidney failure
O10.211	Pre-existing hypertensive chronic kidney disease comp pregnancy, first trimester
O10.212	Pre-existing hypertensive chronic kidney disease comp pregnancy, second trimester
O10.213	Pre-existing hypertensive chronic kidney disease comp pregnancy, third trimester
O10.219	Pre-existing hypertensive chronic kidney disease comp pregnancy, unspecified trimester
O10.22	Pre-existing hypertensive chronic kidney disease comp childbirth
O10.23	Pre-existing hypertensive chronic kidney disease comp the puerperium
O10.311	Pre-existing hypertensive heart and chronic kidney disease comp pregnancy, first trimester
O10.312	Pre-existing hypertensive heart and chr kidney dis comp pregnancy, second trimester
O10.313	Pre-existing hypertensive heart and chr kidney dis comp pregnancy, third trimester
O10.319	Pre-existing hypertensive heart and chr kidney dis comp pregnancy, unspecified trimester
O10.32	Pre-existing hypertensive heart and chronic kidney disease comp childbirth
O10.33	Pre-existing hypertensive heart and chr kidney disease complicating the puerperium
O23.00	Infections of kidney in pregnancy, unspecified trimester
O23.01	Infections of kidney in pregnancy, first trimester
O23.02	Infections of kidney in pregnancy, second trimester
O23.03	Infections of kidney in pregnancy, third trimester
O86.21	Infection of kidney following delivery
O90.4	Postpartum acute kidney failure
Q61.2	Polycystic kidney, adult type
Q61.3	Polycystic kidney, unspecified
Q61.5	Medullary cystic kidney
Q61.8	Other cystic kidney diseases
Q61.9	Cystic kidney disease, unspecified
R94.4	Abnormal results of kidney function studies
S3.7001A	Unspecified injury of right kidney, initial encounter
S37.001D	Unspecified injury of right kidney, subsequent encounter
S37.001S	Unspecified injury of right kidney, sequela
S37.002A	Unspecified injury of left kidney, initial encounter
S37.002D	Unspecified injury of left kidney, subsequent encounter
S37.002S	Unspecified injury of left kidney, sequela
S37.009A	Unspecified injury of unspecified kidney, initial encounter
S37.009D	Unspecified injury of unspecified kidney, subs encounter
S37.009S	Unspecified injury of unspecified kidney, sequela
S37.011A	Minor contusion of right kidney, initial encounter
S37.011D	Minor contusion of right kidney, subsequent encounter
S37.011S	Minor contusion of right kidney, sequela
S37.012A	Minor contusion of left kidney, initial encounter
S37.012D	Minor contusion of left kidney, subsequent encounter
S37.012S	Minor contusion of left kidney, sequela
S37.019A	Minor contusion of unspecified kidney, initial encounter
S37.019D	Minor contusion of unspecified kidney, subsequent encounter
S37.019S	Minor contusion of unspecified kidney, sequela
S37.021A	Major contusion of right kidney, initial encounter
S37.021D	Major contusion of right kidney, subsequent encounter
S37.021S	Major contusion of right kidney, sequela
S37.022A	Major contusion of left kidney, initial encounter
S37.022D	Major contusion of left kidney, subsequent encounter
S37.022S	Major contusion of left kidney, sequela
S37.029A	Major contusion of unspecified kidney, initial encounter
S37.029D	Major contusion of unspecified kidney, subsequent encounter
S37.029S	Major contusion of unspecified kidney, sequela
S37.031A	Laceration of right kidney, unspecified degree, initial encounter
S37.031D	Laceration of right kidney, unspecified degree, subsequent encounter
S37.031S	Laceration of right kidney, unspecified degree, sequela
S37.032A	Laceration of left kidney, unspecified degree, initial encounter
S37.032D	Laceration of left kidney, unspecified degree, subsequent encounter
S37.032S	Laceration of left kidney, unspecified degree, sequela
S37.039A	Laceration of unspecified kidney, unspecified degree, initial encounter
S37.039D	Laceration of unspecified kidney, unspecified degree, subsequent encounter
S37.039S	Laceration of unspecified kidney, unspecified degree, sequela
S37.041A	Minor laceration of right kidney, initial encounter
S37.041D	Minor laceration of right kidney, subsequent encounter
S37.041S	Minor laceration of right kidney, sequela
S37.042A	Minor laceration of left kidney, initial encounter
S37.042D	Minor laceration of left kidney, subsequent encounter
S37.042S	Minor laceration of left kidney, sequela
S37.049A	Minor laceration of unspecified kidney, initial encounter
S37.049D	Minor laceration of unspecified kidney, subsequent encounter
S37.049S	Minor laceration of unspecified kidney, sequela
S37.051A	Moderate laceration of right kidney, initial encounter
S37.051D	Moderate laceration of right kidney, subsequent encounter
S37.051S	Moderate laceration of right kidney, sequela
S37.052A	Moderate laceration of left kidney, initial encounter
S37.052D	Moderate laceration of left kidney, subsequent encounter
S37.052S	Moderate laceration of left kidney, sequela
S37.059A	Moderate laceration of unspecified kidney, initial encounter
S37.059D	Moderate laceration of unspecified kidney, subsequent encounter
S37.059S	Moderate laceration of unspecified kidney, sequela
S37.061A	Major laceration of right kidney, initial encounter
S37.061D	Major laceration of right kidney, subsequent encounter
S37.061S	Major laceration of right kidney, sequela
S37.062A	Major laceration of left kidney, initial encounter
S37.062D	Major laceration of left kidney, subsequent encounter
S37.062S	Major laceration of left kidney, sequela
S37.069A	Major laceration of unspecified kidney, initial encounter
S37.069D	Major laceration of unspecified kidney, subsequent encounter
S37.069S	Major laceration of unspecified kidney, sequela
S37.091A	Other injury of right kidney, initial encounter
S37.091D	Other injury of right kidney, subsequent encounter
S37.091S	Other injury of right kidney, sequela
S37.092A	Other injury of left kidney, initial encounter
S37.092D	Other injury of left kidney, subsequent encounter
S37.092S	Other injury of left kidney, sequela
S37.099A	Other injury of unspecified kidney, initial encounter
S37.099D	Other injury of unspecified kidney, subsequent encounter
S37.099S	Other injury of unspecified kidney, sequela
T86.10	Unspecified complication of kidney transplant
T86.11	Kidney transplant rejection
T86.12	Kidney transplant failure
T86.13	Kidney transplant infection
T86.19	Other complication of kidney transplant

Liver

K70.0	Alcoholic fatty liver
K70.10	Alcoholic hepatitis without ascites
K70.11	Alcoholic hepatitis with ascites
K70.2	Alcoholic fibrosis and sclerosis of liver
K70.30	Alcoholic cirrhosis of liver without ascites
K70.31	Alcoholic cirrhosis of liver with ascites
K70.40	Alcoholic hepatic failure without coma
K70.41	Alcoholic hepatic failure with coma
K70.9	Alcoholic liver disease, unspecified
K71.0	Toxic liver disease with cholestasis
K71.10	Toxic liver disease with hepatic necrosis, without coma
K71.11	Toxic liver disease with hepatic necrosis, with coma
K71.2	Toxic liver disease with acute hepatitis
K71.3	Toxic liver disease with chronic persistent hepatitis
K71.4	Toxic liver disease with chronic lobular hepatitis
K71.50	Toxic liver disease with chronic active hepatitis without ascites
K71.51	Toxic liver disease with chronic active hepatitis with ascites
K71.6	Toxic liver disease with hepatitis, not elsewhere classified
K71.7	Toxic liver disease with fibrosis and cirrhosis of liver
K71.8	Toxic liver disease with other disorders of liver
K71.9	Toxic liver disease, unspecified
K72.00	Acute and subacute hepatic failure without coma
K72.01	Acute and subacute hepatic failure with coma
K72.10	Chronic hepatic failure without coma
K72.11	Chronic hepatic failure with coma
K72.90	Hepatic failure, unspecified without coma
K72.91	Hepatic failure, unspecified with coma
K73.0	Chronic persistent hepatitis, not elsewhere classified
K73.1	Chronic lobular hepatitis, not elsewhere classified
K73.2	Chronic active hepatitis, not elsewhere classified
K73.8	Other chronic hepatitis, not elsewhere classified
K73.9	Chronic hepatitis, unspecified
K74.60	Unspecified cirrhosis of liver
K74.69	Other cirrhosis of liver
K75.0	Abscess of liver
K75.89	Other specified inflammatory liver diseases
K75.9	Inflammatory liver disease, unspecified
K76.0	Fatty (change of) liver, not elsewhere classified
K76.1	Chronic passive congestion of liver
K76.2	Central hemorrhagic necrosis of liver
K76.3	Infarction of liver
K76.89	Other specified diseases of liver
K76.9	Liver disease, unspecified
K77	Liver disorders in diseases classified elsewhere
O26.611	Liver and biliary tract disorders in pregnancy, first trimester
O26.612	Liver and biliary tract disorders in pregnancy, second trimester
O26.613	Liver and biliary tract disorders in pregnancy, third trimester
O26.619	Liver and biliary tract disorders in pregnancy, unspecified trimester
O26.62	Liver and biliary tract disorders in childbirth
O26.63	Liver and biliary tract disorders in the puerperium
Q44.6	Cystic disease of liver
Q44.7	Other congenital malformations of liver
R94.5	Abnormal results of liver function studies
S36.112A	Contusion of liver, initial encounter
S36.112D	Contusion of liver, subsequent encounter
S36.112S	Contusion of liver, sequela
S36.113A	Laceration of liver, unspecified degree, initial encounter
S36.113D	Laceration of liver, unspecified degree, subs encounter
S36.113S	Laceration of liver, unspecified degree, sequela
S36.114A	Minor laceration of liver, initial encounter
S36.114D	Minor laceration of liver, subsequent encounter
S36.114S	Minor laceration of liver, sequela
S36.115A	Moderate laceration of liver, initial encounter
S36.115D	Moderate laceration of liver, subsequent encounter
S36.115S	Moderate laceration of liver, sequela
S36.116A	Major laceration of liver, initial encounter
S36.116D	Major laceration of liver, subsequent encounter
S36.116S	Major laceration of liver, sequela
S36.118A	Other injury of liver, initial encounter
S36.118D	Other injury of liver, subsequent encounter
S36.118S	Other injury of liver, sequela
S36.119A	Unspecified injury of liver, initial encounter
S36.119D	Unspecified injury of liver, subsequent encounter
S36.119S	Unspecified injury of liver, sequela
T86.40	Unspecified complication of liver transplant
T86.41	Liver transplant rejection
T86.42	Liver transplant failure
T86.43	Liver transplant infection
T86.49	Other complications of liver transplant

Opioid Use/Abuse

F11.10	Opioid abuse, uncomplicated
F11.11	Opioid abuse, in remission
F11.120	Opioid abuse with intoxication, uncomplicated
F11.121	Opioid abuse with intoxication delirium
F11.122	Opioid abuse with intoxication with perceptual disturbance
F11.129	Opioid abuse with intoxication, unspecified
F11.13	Opioid abuse with withdrawal
F11.14	Opioid abuse with opioid-induced mood disorder
F11.150	Opioid abuse with opioid-induced psychotic disorder with delusions
F11.151	Opioid abuse with opioid-induced psychotic disorder with hallucinations
F11.159	Opioid abuse with opioid-induced psychotic disorder, unspecified
F11.181	Opioid abuse with opioid-induced sexual dysfunction
F11.182	Opioid abuse with opioid-induced sleep disorder
F11.188	Opioid abuse with other opioid-induced disorder
F11.19	Opioid abuse with unspecified opioid-induced disorder
F11.20	Opioid dependence, uncomplicated
F11.21	Opioid dependence, in remission
F11.220	Opioid dependence with intoxication, uncomplicated
F11.221	Opioid dependence with intoxication delirium
F11.222	Opioid dependence with intoxication with perceptual disturbance
F11.229	Opioid dependence with intoxication, unspecified
F11.23	Opioid dependence with withdrawal
F11.24	Opioid dependence with opioid-induced mood disorder
F11.250	Opioid depend with opioid-induced psychotic disorder with delusions
F11.251	Opioid depend with opioid-induced psychotic disorder with hallucinations
F11.259	Opioid dependence with opioid-induced psychotic disorder, unspecified
F11.281	Opioid dependence with opioid-induced sexual dysfunction
F11.282	Opioid dependence with opioid-induced sleep disorder
F11.288	Opioid dependence with other opioid-induced disorder
F11.29	Opioid dependence with unspecified opioid-induced disorder
F11.90	Opioid use, unspecified, uncomplicated
F1191	Opioid use, unspecified, in remission
F11.920	Opioid use, unspecified with intoxication, uncomplicated
F11.921	Opioid use, unspecified with intoxication delirium
F11.922	Opioid use, unspecified with intoxication with perceptual disturbance
F11.929	Opioid use, unspecified with intoxication, unspecified
F11.93	Opioid use, unspecified with withdrawal
F11.94	Opioid use, unspecified with opioid-induced mood disorder
F11.950	Opioid use, unspecified with opioid-induced psych disorder with delusions
F11.951	Opioid use, unspecified with opioid-induced psych disorder with hallucinations
F11.959	Opioid use, unspecified with opioid-induced psychotic disorder, unspecified
F11.981	Opioid use, unspecified with opioid-induced sexual dysfunction
F11.982	Opioid use, unspecified with opioid-induced sleep disorder
F11.988	Opioid use, unspecified with other opioid-induced disorder
F11.99	Opioid use, unspecified with unspecified opioid-induced disorder
T40.0X1A	Poisoning by opium, accidental (unintentional), initial encounter
T40.0X1D	Poisoning by opium, accidental (unintentional), subsequent encounter
T40.0X1S	Poisoning by opium, accidental (unintentional), sequela
T40.0X2A	Poisoning by opium, intentional self-harm, initial encounter
T40.0X2D	Poisoning by opium, intentional self-harm, subsequent encounter
T40.0X2S	Poisoning by opium, intentional self-harm, sequela
T40.0X3A	Poisoning by opium, assault, initial encounter
T40.0X3D	Poisoning by opium, assault, subsequent encounter
T40.0X3S	Poisoning by opium, assault, sequela
T40.0X4A	Poisoning by opium, undetermined, initial encounter
T40.0X4D	Poisoning by opium, undetermined, subsequent encounter
T40.0X4S	Poisoning by opium, undetermined, sequela
T40.0X5A	Adverse effect of opium, initial encounter
T40.0X5D	Adverse effect of opium, subsequent encounter
T40.0X5S	Adverse effect of opium, sequela
T40.0X6A	Underdosing of opium, initial encounter
T40.0X6D	Underdosing of opium, subsequent encounter
T40.0X6S	Underdosing of opium, sequela
T40.2X1A	Poisoning by other opioids, accidental (unintentional), initial encounter
T40.2X1D	Poisoning by other opioids, accidental (unintentional), subsequent encounter
T40.2X1S	Poisoning by other opioids, accidental, sequela
T40.2X2A	Poisoning by other opioids, intentional self-harm, initial encounter
T40.2X2D	Poisoning by other opioids, intentional self-harm, subsequent encounter
T40.2X2S	Poisoning by other opioids, intentional self-harm, sequela
T40.2X3A	Poisoning by other opioids, assault, initial encounter
T40.2X3D	Poisoning by other opioids, assault, subsequent encounter
T40.2X3S	Poisoning by other opioids, assault, sequela
T40.2X4A	Poisoning by other opioids, undetermined, initial encounter
T40.2X4D	Poisoning by other opioids, undetermined, subs encounter
T40.2X4S	Poisoning by other opioids, undetermined, sequela
T40.2X5A	Adverse effect of other opioids, initial encounter
T40.2X5D	Adverse effect of other opioids, subsequent encounter
T40.2X5S	Adverse effect of other opioids, sequela
T40.2X6A	Underdosing of other opioids, initial encounter
T40.2X6D	Underdosing of other opioids, subsequent encounter
T40.2X6S	Underdosing of other opioids, sequela

Pain

F45.41	Pain disorder exclusively related to psychological factors
F45.42	Pain disorder with related psychological factors
G89.0	Central pain syndrome
G89.11	Acute pain due to trauma
G89.18	Other acute postprocedural pain
G89.21	Chronic pain due to trauma
G89.22	Chronic post-thoracotomy pain
G89.28	Other chronic postprocedural pain
G89.29	Other chronic pain
G89.3	Neoplasm-related pain (acute) (chronic)
G89.4	Chronic pain syndrome
G90.50	Complex regional pain syndrome I, unspecified
G90.511	Complex regional pain syndrome I of right upper limb
G90.512	Complex regional pain syndrome I of left upper limb
G90.513	Complex regional pain syndrome I of upper limb, bilateral
G90.519	Complex regional pain syndrome I of unspecified upper limb
G90.521	Complex regional pain syndrome I of right lower limb
G90.522	Complex regional pain syndrome I of left lower limb
G90.523	Complex regional pain syndrome I of lower limb, bilateral
G90.529	Complex regional pain syndrome I of unspecified lower limb
G90.59	Complex regional pain syndrome I of other specified site
I83.811	Varicose veins of right lower extremity with pain
I83.812	Varicose veins of left lower extremity with pain
I83.813	Varicose veins of bilateral lower extremities with pain
I83.819	Varicose veins of unspecified lower extremity with pain
M25.50	Pain in unspecified joint
M25.51	Pain in shoulder
M25.511	Pain in right shoulder
M25.512	Pain in left shoulder
M25.519	Pain in unspecified shoulder
M25.521	Pain in right elbow
M25.522	Pain in left elbow
M25.529	Pain in unspecified elbow
M25.531	Pain in right wrist
M25.532	Pain in left wrist
M25.539	Pain in unspecified wrist
M25.541	Pain in joints of right hand
M25.542	Pain in joints of left hand
M25.549	Pain in joints of unspecified hand
M25.551	Pain in right hip
M25.552	Pain in left hip
M25.559	Pain in unspecified hip
M25.561	Pain in right knee
M25.562	Pain in left knee
M25.569	Pain in unspecified knee
M25.571	Pain in right ankle and joints of right foot
M25.572	Pain in left ankle and joints of left foot
M25.579	Pain in unspecified ankle and joints of unspecified foot
M25.59	Pain in other specified joint
M54.5	Low back pain
M54.50	Low back pain, unspecified
M54.51	Vertebrogenic low back pain
M54.59	Other low back pain
M54.6	Pain in thoracic spine
M79.601	Pain in right arm
M79.602	Pain in left arm
M79.603	Pain in arm, unspecified
M79.604	Pain in right leg
M79.605	Pain in left leg
M79.606	Pain in leg, unspecified
M79.609	Pain in unspecified limb
M79.621	Pain in right upper arm
M79.622	Pain in left upper arm
M79.629	Pain in unspecified upper arm
M79.631	Pain in right forearm
M79.632	Pain in left forearm
M79.639	Pain in unspecified forearm
M79.641	Pain in right hand
M79.642	Pain in left hand
M79.643	Pain in unspecified hand
M79.644	Pain in right finger(s)
M79.645	Pain in left finger(s)
M79.646	Pain in unspecified finger(s)
M79.651	Pain in right thigh
M79.652	Pain in left thigh
M79.659	Pain in unspecified thigh
M79.661	Pain in right lower leg
M79.662	Pain in left lower leg
M79.669	Pain in unspecified lower leg
M79.671	Pain in right foot
M79.672	Pain in left foot
M79.673	Pain in unspecified foot
M79.674	Pain in right toe(s)
M79.675	Pain in left toe(s)
M79.676	Pain in unspecified toe(s)
R39.82	Chronic bladder pain
R52	Pain, unspecified
